# Are Women’s Empowerment and Income Inequality Associated with Excess Weight in Latin American Cities?

**DOI:** 10.1007/s11524-022-00689-5

**Published:** 2022-11-10

**Authors:** Natalia Tumas, Santiago Rodríguez López, Mónica Mazariegos, Ana Ortigoza, Cecilia Anza Ramírez, Carolina Pérez Ferrer, Kari Moore, Goro Yamada, Mariana Carvalho Menezes, Olga L. Sarmiento, Juan M. Pericàs, Francesc Belvis Costes, Mariana Lazo, Joan Benach

**Affiliations:** Department of Political and Social Sciences, Research Group on Health Inequalities, Environment, Employment Conditions Knowledge Network (GREDS-EMCONET), Universitat Pompeu Fabra, Barcelona, Spain; Johns Hopkins University - Pompeu Fabra University Public Policy Center (UPF-BSM), Universitat Pompeu Fabra, Barcelona, Spain; Centro de Investigaciones y Estudios sobre Cultura y Sociedad, Consejo Nacional de Investigaciones Científicas y Técnicas (CONICET) y Universidad Nacional de Córdoba, Córdoba, Argentina; Centro de Investigaciones y Estudios sobre Cultura y Sociedad, Consejo Nacional de Investigaciones Científicas y Técnicas (CONICET) y Universidad Nacional de Córdoba, Córdoba, Argentina; Facultad de Ciencias Exactas, Físicasy Naturales, Universidad Nacional de Córdoba, Córdoba, Argentina; INCAP Research Center for the Prevention of Chronic Diseases (CIIPEC), Institute of Nutrition of Central America and Panama (INCAP), Guatemala City, Guatemala; Urban Health Collaborative, Dornsife School of Public Health, Drexel University, Philadelphia, USA; CRONICAS Centre of Excellence in Chronic Diseases, Universidad PeruanaCayetano Heredia, Lima, Perú; National Institute of Public Health, Cuernavaca, Mexico; Urban Health Collaborative, Dornsife School of Public Health, Drexel University, Philadelphia, USA; Urban Health Collaborative, Dornsife School of Public Health, Drexel University, Philadelphia, USA; Universidade Federal de Ouro Preto, Ouro Preto, Brazil; School of Medicine, Universidad de los Andes, Bogotá, Colombia; Department of Political and Social Sciences, Research Group on Health Inequalities, Environment, Employment Conditions Knowledge Network (GREDS-EMCONET), Universitat Pompeu Fabra, Barcelona, Spain; Johns Hopkins University - Pompeu Fabra University Public Policy Center (UPF-BSM), Universitat Pompeu Fabra, Barcelona, Spain; Liver Unit, Internal Medicine Department, Vall d’Hebron Institute for Research, Vall d’Hebron University Hospital, Barcelona, Spain; Centro de Investigación Biomédica en Red de EnfermedadesHepáticas y Digestivas (CIBERehd), Madrid, Spain; Johns Hopkins University - Pompeu Fabra University Public Policy Center (UPF-BSM), Universitat Pompeu Fabra, Barcelona, Spain; Urban Health Collaborative, Dornsife School of Public Health, Drexel University, Philadelphia, USA; Department of Political and Social Sciences, Research Group on Health Inequalities, Environment, Employment Conditions Knowledge Network (GREDS-EMCONET), Universitat Pompeu Fabra, Barcelona, Spain; Johns Hopkins University - Pompeu Fabra University Public Policy Center (UPF-BSM), Universitat Pompeu Fabra, Barcelona, Spain; Ecological Humanities Research Group (GHECO), Universidad Autónoma, Madrid, Spain

**Keywords:** Women’s empowerment, Income inequality, Overweight, Obesity, Cities, Latin America

## Abstract

While income gradients and gender inequalities in excess weight have been noted elsewhere, data from Latin American cities is lacking. We analyzed gender-specific associations between city-level women’s empowerment and income inequality with individual-level overweight/obesity, assessing how these associations vary by individual education or living conditions within cities in Latin America. Data came from national surveys and censuses, and was compiled by the SALURBAL project (Urban Health in Latin America). The sample included 79,422 individuals (58.0% women), living in 538 sub-cities, 187 cities, and 8 countries. We used gender-stratified Poisson multilevel models to estimate the Prevalence Rate Ratios (PRR) for overweight/obesity (body mass index ≥ 25 kg/m^2^) per a unit change in city-level women’s empowerment (proxied by a score that measures gender inequalities in employment and education) and income inequality (proxied by income-based Gini coefficient). We also tested whether individual education or sub-city living conditions modified such associations. Higher city labor women’s empowerment (in women) and higher city Gini coefficient (in men) were associated with a lower prevalence of overweight/obesity (PRR = 0.97 (95%CI 0.94, 0.99) and PRR = 0.94 (95%CI 0.90, 0.97), respectively). The associations varied by individual education and sub-city living conditions. For labor women’s empowerment, we observed weakened associations towards the null effect in women with lower education and in residents of sub-cities with worse living conditions (men and women). For the Gini coefficient, the association was stronger among men with primary education, and a negative association was observed in women with primary education. Our findings highlight the need for promoting equity-based policies and interventions to tackle the high prevalence of excess weight in Latin American cities.

## Introduction

Excess weight (overweight/obesity), urbanization, and social and gender inequalities are closely interrelated growing global issues. Approximately half of the world’s population lives in urban areas [1], and about 39% of the world’s adult population was living with overweight or obesity in 2015 [2]. In parallel, social and gender inequalities are one of the most important determinants of population health [3–5]. Indeed, excess weight is more prevalent among more unequal societies [6], and income gradients and gender inequalities in overweight/obesity have been noted [7, 8]. In addition, obesity and social inequalities are higher in large urbanized areas [4, 9, 10].

Gender-based inequalities in overweight/obesity are an important concern in improving gender equity and population health [11]. Women’s empowerment can be defined as efforts to address gender inequalities in education and employment which then lead to improved access and use of resources that allow women to develop their potential [12]. The role of women’s empowerment to tackle malnutrition and improving nutritional outcomes has been reported [12, 13], but the evidence on overweight/obesity is inconclusive, and this relationship is not fully understood. On the one hand, a study including several countries showed that women’s empowerment was associated with a lower obesity prevalence [14] and a higher dietary diversity among women in Ghana (but not with body mass index (BMI)) [15]. On the other hand, evidence from global data showed that increasing women’s empowerment appeared to be associated with increases in BMI among women [16]. Women empowerment might reduce the social pressure to comply with body-image stereotypes, as well as reduce home cooking—traditionally carried out by women—easing a greater consumption of ultraprocessed high-calorie products [11, 17, 18]. However, women’s empowerment might also contribute to a lower probability of overweight/obesity through improvements in nutritional education [14] and lower interest in maternity, as pregnancies are associated with increments in adiposity in women [19]. A potential explanation for the conflicting results is that women’s empowerment is context-specific and its operationalization varies widely, making comparisons across studies challenging.

Evidence of the association between area-income inequality and excess weight is also inconsistent. Previous research reported that income inequality is related to obesity, as inequality may determine obesogenic environments that influence individuals’ choices towards less healthy dietary patterns and lifestyles [4, 14], and the psychosocial impacts of living in a more hierarchical society might lead to excess weight [20]. However, some evidence showed that income inequality was also negatively associated with excess weight [21, 22], although the mechanisms are less clear. Research from high-income countries showed that higher income inequality was positively correlated with BMI or obesity [8, 20], but others suggested an association with lower obesity in men [23]. In low- and middle-income countries (LMICs), the evidence is more limited. Low-income inequality was linked to faster increments of overweight rates in women from lower socioeconomic groups [21]. Particularly in Latin America, it was described a negative association with BMI and overweight/obesity among Mexican women [22].

The rising prevalence of overweight/obesity and related-risk behaviors has not been similar between and within regions and countries [24, 25]. In high-income countries, the prevalence began to attenuate in the past decade, while rising trends are likely to continue in LMICs, where more than half of people with obesity live [25, 26]. Furthermore, research shows that as a country’s development increases, the burden of obesity shifts toward the lower socioeconomic groups, and that this pattern is more noticeable among women [25, 26]. Less is known about how social and gender inequalities shape the pattern of overweight/obesity between and within cities, especially in LMICs. Particularly in Latin America, excess weight affects approximately 50% of the adult population, and increasing trends are projected, mainly among women [27, 28]. Additionally, Latin America is among the most urbanized regions worldwide, with fast-growing rates: the urban population grew from 40% in 1950 to 80% in 2015 [1]. At the same time, this is one of the most unequal regions, including more than half of the most unequal cities [29, 30]. Gender inequalities also characterize the region: poverty, difficulties in schooling, and low levels of participation in the labor force, among others, affect women predominantly [31, 32]. In women, there are large educational inequalities in obesity independent of city socioeconomic development, while in men, education gradients were observed only in cities with lower socioeconomic development [33]. Social contexts within Latin American cities are heterogeneous [30, 34] as they vary in socioeconomic conditions, the degree of poverty and social and gender inequalities, ethnic composition, among others, and may play a role in the social patterning of overweight/obesity as well [33, 35].

Given the concomitant features of high urbanization rates, high levels of social and gender inequalities, and high prevalence of overweight/obesity, Latin America is a unique scenario for studying the urban social and gender inequalities in overweight/obesity. To the best of our knowledge, no prior study has assessed the association between women’s empowerment and income inequality with overweight/obesity in Latin American cities. Thus, the aims of this study are to analyze the association between (1) city-level women’s empowerment and (2) income inequality with overweight/obesity by gender and (3) to assess how these associations vary by individual education or by the living conditions within cities in Latin America. We hypothesize that individuals living in cities with greater women’s empowerment have a lower prevalence of overweight/obesity compared to those from cities with lower women’s empowerment, through improved nutritional education and reduced interest in maternity. Furthermore, individuals living in cities with greater income inequality have a higher prevalence of overweight/obesity compared to those from less unequal cities, mainly due to the greater exposure to obesogenic environments in highly unequal contexts. We expect these associations to be stronger among women. Last, these associations are modified by individual education and by the living conditions within the cities.

## Methods

### Sample

This cross-sectional study was developed using data compiled by the SALURBAL project (Urban Health in Latin America; “Salud Urbana en América Latina”) [36], a collaborative multinational project that examines drivers of health in Latin American cities of more than 100,000 inhabitants [30]. We analyzed harmonized data from health surveys and population censuses ranging from 2002 to 2017 in: Argentina, Brazil, Chile, Colombia, El Salvador, Guatemala, Mexico, and Peru (for details on the years of surveys and census in each country, see Table SI, Supplementary material). The analytical sample included a total of 79,422 individuals (58.0% women), living in 187 cities and 538 subcities (administrative units within cities). Sub-cities were defined as *municipios*, *comunas*, *departamentos*, or similar units depending on the country. There was a median of 708 individuals per city (interquartile range (IQR), 422–1,114), 474 individuals per sub-city (IQR, 135–819), and 5 sub-cities per city (IQR, 2–35).

### Outcome

We included excess weight (which encompasses overweight/obesity, BMI ≥ 25 kg/m^2^) as the outcome at the individual level. For all countries except Argentina, BMI was calculated based on standardized measures of weight and height. For Argentina, we used self-reported weight and height, as the survey did not include measured weight and height.

### Exposures

We included two exposures at the city level: labor women’s empowerment and the Gini coefficient. In brief, labor women’s empowerment measures account for gender inequalities in education and employment [37, 38]. We operationalized labor women’s empowerment by means of a score developed using national census data on employment and education disaggregated by cities and sex and derived by principal component analysis [38]. The data used to develop the score included: the ratio of the female-to-male proportion of the population aged 25 or older who completed secondary education or above, the ratio of the female-to-male proportion of the population aged 25 or older who completed university or above, the labor force participation rate among the female population 15 years or above, and the ratio of the labor force participation rate among females to the labor force participation rate among males (15 years or older). Higher score values indicate greater participation of women in the labor force and greater education of women relative to men. We proxied income inequality by the income-based Gini coefficient, calculated based on census information or national household surveys. The Gini coefficient is a well-known summary measure of the distribution of per-capita household income in the city, with higher values indicating greater income inequality. We limited the sample to the cities with available data on income-based Gini coefficient (187 of 232 of the SALURBAL cities included in this study).

### Effect Modifiers

We evaluated individual education and sub-city living conditions as potential effect modifiers. Individual educational attainment was categorized as: less than primary, primary, secondary, and university and obtained from the same health survey data used to define the outcome. Sub-city living conditions were measured using a composite index based on census indicators of general living conditions, including the percentage of households with piped water inside the dwelling, the percentage of households with overcrowding (3 or more people per room, inverted), and the percentage of the population aged 15–17 attending school [34]. Higher score values correspond to better living conditions.

### Covariates

We adjusted the analyses by age (quadratic term, given the non-linear association between age and overweight/obesity) [39], city population size (because previous studies showed links between population size and overweight/obesity) [40], city GDP per capita (in order to test to what extent labor women’s empowerment and Gini coefficient were associated with overweight/obesity beyond GDP of cities) [21], and country (in order to control for unmeasured confounding effects). We stratified the analysis by gender (using self-reported sex female/male), considering that the effect of social indicators on overweight/obesity differed by gender [33, 41]. For details on the study variables and levels of analysis, see Table SII, Supplementary material.

### Statistical Analysis

We described the distribution of individual-, subcity-, and city-level characteristics by presence or absence of overweight/obesity. We estimated age-adjusted percentages of overweight/obesity (through direct standardization, using the pooled sample age distribution) by quartiles of labor women’s empowerment and Gini coefficient and by gender.

We estimated gender-specific associations between overweight/obesity and the exposures of interest, using three-level Poisson models with robust variance estimation and random intercepts for sub-cities and cities. We used two models: (i) the *adjusted model* included individual age (quadratic), individual education (categories), sub-city living conditions (z scores), city population size (z scores), city GDP per-capita (z scores), labor women’s empowerment (z scores) (*adjusted model a*)/Gini coefficient (z scores) (*adjusted model b*)—separately—and countries (categories) as fixed effects; (ii) the *interaction model* included interaction terms (labor women’s empowerment/Gini coefficient*individual education; labor women’s empowerment/Gini coefficient* sub-city living conditions) and all the adjustment variables. Continuous variables were standardized in all models, using the pooled sample (men and women). The z-score has a mean of zero and a standard deviation of one and indicates how many standard deviations the variable is from the mean. It is obtained by subtracting the mean from each observation and then dividing the difference by the standard deviation. Statistical analysis was performed with Stata 15 [42] and R 4.1.0 [43].

## Results

A higher percentage of men (63.8%) had overweight/obesity compared to women (60.4%), and individuals with overweight/obesity were older compared to those without this condition (Table 1). Overweight/obesity was more frequent in individuals with lower educational level and in sub-cities with worse living conditions. In addition, respondents with over-weight/obesity were more likely to live in cities with higher GDP and with lower labor women’s empowerment. The city Gini coefficient was similar among the individuals with and without overweight/obesity, although differences were statistically significant.

**Fig. 1:**
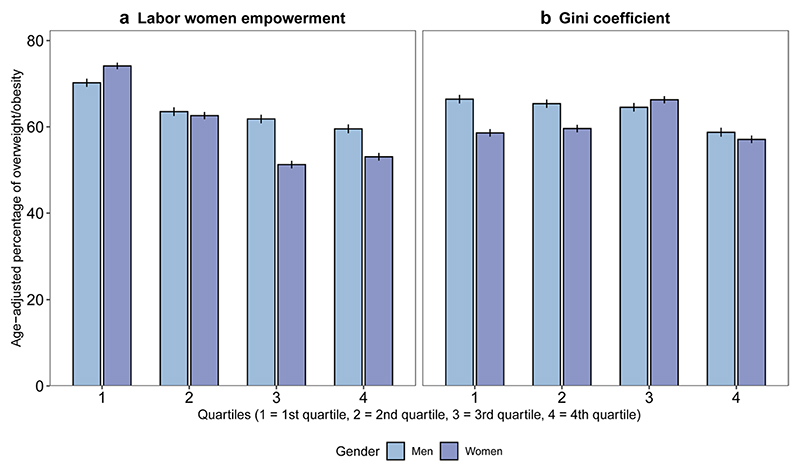
Age-adjusted percentage of overweight/obesity by quartiles of city. Gini coefficient and labor women’s empowerment and gender in 187 Latin American cities. SALURBAL study. 95% confidence intervals are shown in vertical lines

Overweight/obesity was patterned by city-level labor women’s empowerment, with a lower proportion of overweight/obesity in cities with higher labor women’s empowerment (more evident in women) (Fig. 1). Patterns by the Gini coefficient were less clear, although the age-adjusted percentage of over-weight/obesity in men and women was lowest at the highest city income inequality quartile (Fig. 1).

Table 2 shows the adjusted prevalence rate ratios (PRRs) of overweight/obesity associated with city labor women’s empowerment (adjusted model a) and city Gini coefficient (adjusted model b) for women and men separately. After adjusting for individual characteristics, city GDP per-capita, city population size, and country, both higher city labor women’s empowerment (in women) and higher city Gini coefficient (in men) were associated with a lower prevalence of overweight/obesity (PRR = 0.97 (95% CI 0.94, 0.99) and PRR = 0.94 (95% CI 0.90, 0.97), respectively).

**Fig. 2:**
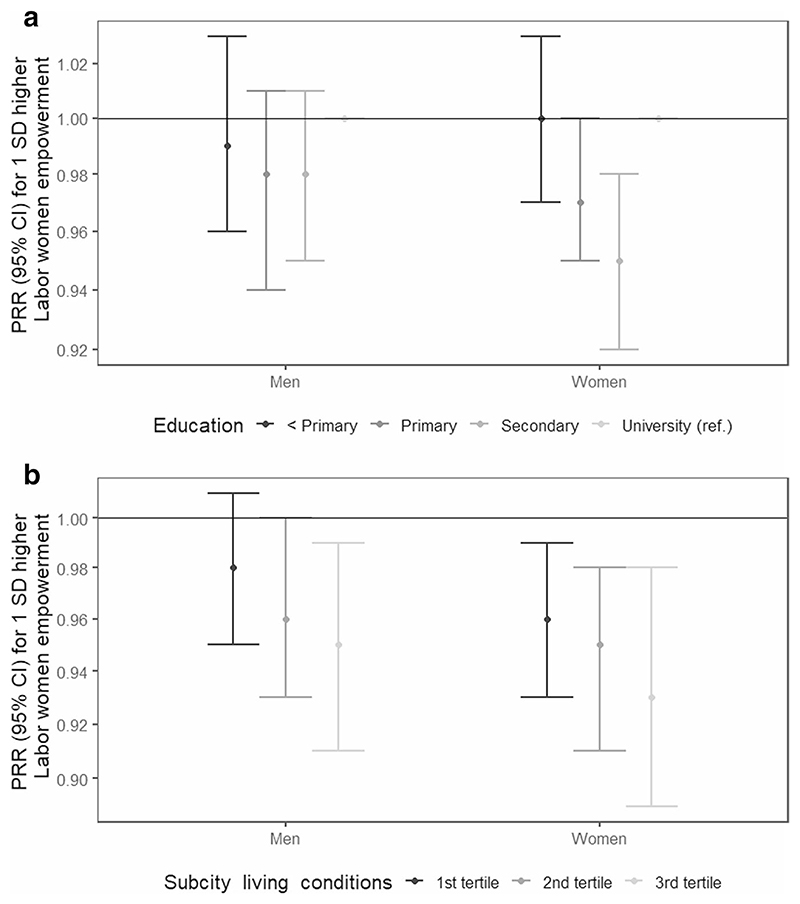
Adjusted associations between labor women’s empowerment and overweight/obesity, by **a** individual education and **b** sub-city living conditions in men and women in 187 Latin American cities. SALURBAL study. Significant global *p*-values for interactions: < 0.01 for individual education in women; < 0.05 for sub-city living conditions in men

**Fig. 3:**
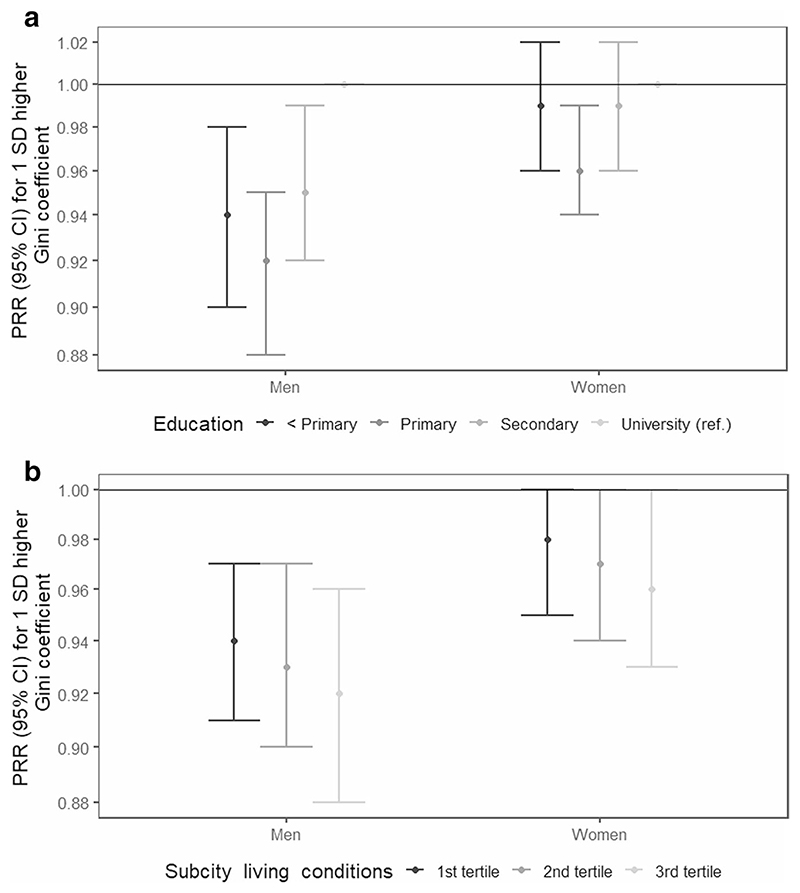
Adjusted associations between Gini coefficient and overweight/obesity, by **a** individual education and **b** sub-city living conditions in men and women in 187 Latin American cities. SALURBAL study. Significant global *p*-values for interactions: < 0.05 for individual education in men and < 0.01 in women

The results of the interaction analyses are shown in Figs. 2a and b and 3a and b. Among women, the association between labor women’s empowerment and overweight/obesity was different across individual education (global p-value for interaction <0.01). The protective association with increased labor women’s empowerment was null for women with less than primary education (Fig. 2a). Similarly, the protective association with increased labor women’s empowerment was weaker among women living in sub-cities with worse living conditions (Fig. 2b). In men, a similar pattern was observed for sub-city living conditions (global p-value for interaction < 0.05) (Fig. 2b). The results of the interaction analyses of the association between the Gini coefficient and over-weight/obesity are shown in Fig. 3a and b. Among men, the negative association between the Gini coefficient and overweight/obesity was different across individual education (global p-value for interaction < 0.05), being stronger among men with lower levels of education, compared to those with university (Fig. 3a). In women, a negative association was only observed among those with primary education (global p-value for interaction < 0.01) (Fig. 3a). Last, associations were similar across tertiles of sub-city living conditions (Fig. 3b).

## Discussion

We analyzed associations between city-level income inequality and labor women’s empowerment with overweight/obesity by gender in a large sample of Latin American cities and examined how these associations vary by individual education or by sub-city living conditions. We identified a lower prevalence of overweight/obesity associated with higher city labor women’s empowerment in women and with higher city income inequality in men, after adjusting for individual-, sub-city-, and city-level factors. Furthermore, the negative association between labor women’s empowerment and overweight/obesity in women varied by individual education and by subcity living conditions, with weakened associations towards the null effect in those with the lowest educational level and in residents of sub-cities with worse living conditions. A similar pattern was observed for sub-city living conditions in men. We also found that individual education modified the association between income inequality and overweight/obesity in men, such that the association was stronger among those with primary education. In women, a negative association was observed among those with primary education.

While there is a relative consensus that women’s empowerment might prevent undernutrition [12], the evidence is scarce and less consistent for excess weight. Aligned with our first hypothesis, we found an inverse association between women’s empowerment and the prevalence of overweight/obesity, only among women. Consistent with our results, a study including almost 160 countries described a lower prevalence of obesity associated with higher women’s empowerment [14]. Contrary to our findings, another study including 190 countries showed that women’s empowerment was associated with increases in BMI over time among women [18], and a recent study including eastern African countries also reported a positive association with women’s BMI [16]. The discrepancy between these findings could be partially explained by other broader regional and country contextual factors related to what women’s empowerment implies for factors linked to overweight/obesity (such as diet and physical activity) and also to differences in social and cultural norms associated with women being more empowered. Also, the different methodological approaches and the scales of the women’s empowerment measures used in each case might contribute to the divergence in the results [7]. Indeed, there are important gaps and heterogeneities in the measurement of women’s empowerment, and the application of existing measures is still limited [12]. We are not aware of any previous research studying the relationship between women’s empowerment and excess weight in Latin America, but prior evidence in cities of the region showed that women’s labor force participation (as a proxy of women’s empowerment) was negatively associated with infant mortality rate, another key population health indicator [38].

Several mechanisms might underlie the inverse associations between women’s empowerment and overweight/obesity. On the one hand, an indirect pathway through employment and higher income, as well as a greater education level, that accompany women’s empowerment [11]. Growing evidence has documented that empowerment leads to better employment conditions, education, political participation, and health [11, 44] and to improved engagement in the prevention of non-communicable diseases, including weight loss [11, 45, 46]. The smoking habit might also play a role, given its association with women empowerment [47] and weight loss [48]. In addition, lower fertility rates at higher levels of education might influence these associations, as pregnancy-related weight gain has emerged as a potential cause of excess adiposity [49]. The latter could be of importance in Latin America because of the elevated adolescent fertility, which is strongly linked with long-term increments in adiposity [19]. We hypothesize that women’s empowerment might decrease over-weight/obesity indirectly by reducing the interest in maternity and therefore cumulative weight gain associated with parity. However, we did not have information about parity to further explore this hypothesis in our study. On the other hand, research suggested that socioeconomic development and women’s changing roles in societies may affect the excess weight underlying behavioral mechanisms [18]. The “modernization theory” states that socioeconomic development leads to domestic nutrition transitions (from low to high caloric diet) and relates the greater unhealthy food intake to increasing income and higher participation of women in the labor force [18, 50, 51]. The latter usually implies less homemade food and greater consumption of high-calorie ultra-processed products [17]. It should be noted that the dominant models of production, the shifts in the agricultural system, and the change in technology, mass media, and urbanization are important drivers of the nutrition transitions [52]. Another proposed mechanism suggests that women’s empowerment might reduce the social pressure that traditionally faces women to align to body size preferences and patterns [11].

We also found an inverse association between city income inequality and the prevalence of over-weight/obesity in men, contrary to what we hypothesized. Similar to our study, greater income inequality was linked to lower obesity rates across counties of New York only in men [23]. Evidence from Mexico also revealed an inverse association with BMI and overweight/obesity among women [22]. Moreover, Jones-Smith et al. [21] in a sample of women from 37 LMICs found that lower income inequality was associated with faster increments of overweight in the lower wealth groups from countries with a higher GDP. In contrast, in a study including 127 low-, middle-, and high-income countries, De Vogli et al. [53] found a positive association between the Gini coefficient and BMI. In another study using country-aggregated data of OECD countries, higher income inequality was linked to a faster increase in obesity prevalence [54]. Evidence from high-income countries showed a positive association between income inequality and the percentage of obesity [20] and BMI [8]. Relative to this, it was suggested that high-income inequality leads to increases in most of the health conditions associated with low social position, including overweight/obesity [55]. Inequality is a scale-specific contextual indicator and it is differently related to social conditions. Thus, different geographic areas considered could contribute to differences in the associations [23].

The evidence about the gender-specific impacts of income inequality on overweight/obesity and the underlying mechanisms is still scarce. The existing literature showed that income inequality has detrimental effects on social cohesion, and also that fastfood outing could be considered a proxy of social capital [22, 56]. Prior research argued that since men tended to have greater non-kin ties in their personal networks than women [57], significant effects of income inequality could be observed in men but not in women. In addition, Prince et al. [58] found higher probability of overweight/obesity associated with a stronger sense of community belonging in men, but no significant impact was evidenced on women. Based on the above, we hypothesize that in contexts of high inequality, men could be more vulnerable than women to the impact of lack of social cohesion on outing unhealthy food intake. However, the pathways to explain the gender differences in these associations are unclear and require further research.

Some explanations have been proposed for the link between higher income inequality and a lower prevalence of overweight/obesity. Clement et al. [22] demonstrated that the main pathways proposed to explain the consequences of income inequality on health outcomes [54] also explain the negative associations between income inequality and excess weight. They showed that income inequality might limit weight gain through a worse quality of sleep (psychological pathway), less fast-food consumption (social capital pathway), and limited access to piped water—through weight loss associated with increased occurrence of infectious diseases and increased physical activity to fetch and carry water (public policy pathway). Additional research is needed to elucidate the mechanisms underlying these associations.

Overall, we also found that the associations between overweight/obesity and women’s empowerment and income inequality were modified by individual education and to a lesser extent by sub-cities living conditions, which is most aligned with our third hypothesis. Specifically, women with less than primary education and individuals living in sub-cities with worse living conditions were less or not benefited by the potential protective effect of women’s empowerment on overweight/obesity. These findings could be linked to the relative deprivation hypothesis, which posits that better area-level social conditions may not imply garnering health benefits and even could be unfavorable for those individuals with lower social positions [55]. We also found that men with primary education living in cities with high levels of income inequality seemed to have even lower prevalence of overweight/obesity. In line with that, Bjorn-strom [59] and Fan et al. [60] in the USA showed that the lowest social position groups from unequal localities can incorporate some health-related behaviors from the high social position groups, leading to better nutritional outcomes for the former.

Our findings have policy implications. We provide evidence suggesting that increasing education and labor force participation among women might be relevant for addressing overweight/obesity in Latin America. This should be acknowledged together with policies aimed to promote healthier lifestyles. Public policies and interventions at the population level should be oriented to improve living and working environments (e.g., through improved access to healthy food) and promote healthy macro policies (regulations and fiscal measures, such as taxes on high-fat and high-sugar food). Also, interventions targeting less educated groups and focusing on socioeconomically disadvantaged areas of cities are recommended to reduce disparities in overweight/obesity.

Strengths of our study include the use of a multi-level harmonized database of national health surveys combined with contextual data on city social characteristics, accounting for the heterogeneity within cities in several Latin American countries. To our knowledge, this is the first study considering women’s empowerment and income inequality indicators at the city level, coupled with information about the sub-city social features, providing a singular perspective on the interplay across urban social gradients in excess weight in Latin America. Overall, our study sheds light on the linkages between important issues that, to date, remain scarcely studied. Future work should further explore the mechanisms behind the associations we observed, and longitudinal research will also contribute to a better understanding of the social and gendered patterning of overweight/obesity in Latin America. Our study has some limitations. We acknowledge that the score used to proxy women’s empowerment was based on census data and built upon employment and education indicators, not reflecting other dimensions of the empowerment of women such as decision-making power and gender-based violence, among others [38, 61]. We did not include in the analysis the total fertility rate due to a lack of data; however, prior research has argued that obesity due to multiparity in women is, at least in part, driven by the social context in which the reproductive rights of women are situated [7]. Prioritizing comparisons across countries, we assumed that women from 15 to 18 years within the work market would represent a higher women’s empowerment, which is arguable. Nevertheless, we expect that the number of women in the workforce within this age range would be rather low. The countries in our study are heterogeneous in the number of native ethnic communities in cities, which may have a higher risk of overweight/obesity because, usually, they are socially excluded, have lower income, and are less empowered [62]. We did not account for this heterogeneity within cities, which may play a role in our findings. Furthermore, city characteristics were retrieved from censuses developed at different years in the countries, frequently not aligned with the health survey years from which we obtained the individual data. This forced us to assume that city characteristics were relatively stable across the years examined. Data on income inequality (income-based Gini coefficient) was not available for all cities, and, e.g., only a single city for Guatemala and Peru could be included. Other limitations were the cross-sectional design, which prevented us from assuming any type of causality, and the use of self-reported weight and height in Argentina, although evidence suggested good agreement between objective and self-reported weight and height [63].

## Conclusion

In summary, we found that the prevalence of over-weight/obesity was negatively associated with women’s empowerment in women and income inequality in men. Our results also suggested that city inequalities differ across educational backgrounds and—to a lesser extent—across the sub-cities living conditions. Overall, these findings highlight the need for promoting equity-based policies and interventions to tackle excess weight in Latin America. Improving women’s empowerment is especially recommended to address the gendered overweight/obesity pandemic in the region.

## Supplementary Material

Supplementary

## Figures and Tables

**Table 1:** Study characteristics by overweight/obesity status in 187 Latin American cities. SALURBAL Study

	Overall sample	Overweight/obesity		
		(BMI ≥ 25 kg/m^2^)		
	(*n* = 79,422)	Yes (*n* = 49,085)	No (*n* = 30,337)	*p*-value*
Gender, %				
Women	58.0	60.4	39.6	< 0.01
Men	42.0	63.8	36.2	
Age, mean (SD)	42.8 (16.9)	44.9 (15.9)	39.3 (17.8)	< 0.01
Individual education, %				
University	13.4	12.4	15.0	< 0.01
Secondary	34.2	31.0	37.9	
Primary	35.2	37.0	32.3	
Less than primary	17.2	18.6	14.8	
Sub-city living conditions, mean (SE)	0.72 (1.96)	0.59 (2.00)	0.91 (1.87)	< 0.01
City labor women’s empowerment, mean (SD)	0.65 (3.03)	0.34 (3.09)	1.15 (2.85)	< 0.01
City Gini coefficient, mean (SD)	0.49 (0.10)	0.49 (0.10)	0.50 (0.10)	< 0.01
City GDP per-capita (USD, thousands), mean (SD)	18.2 (13.6)	18.7 (14.6)	17.5 (11.8)	< 0.01
City population size (millions), mean (SD)	3.7 (5.6)	3.7 (5.7)	3.7 (5.6)	0.93

*SD*, standard deviation; *GDP*, gross domestic product. Sub-city living conditions range from − 9.92 to 3.90 with higher scores corresponding to better conditions; labor women’s empowerment ranges from − 8.20 to 6.28 with higher scores corresponding to higher labor women’s empowerment; Gini coefficient ranges from 0.29 to 0.68 with higher scores corresponding to higher income inequality.

* p-values test the null hypothesis that there is no difference in the proportion (χ^2^) or mean (t-test) of the characteristic between individuals with and without overweight/obesity

**Table 2:** Prevalence rate ratios (95% confidence intervals) of overweight/obesity associated with city characteristics in 187 Latin American cities. SALURBAL study

	PRR (95% CI)	
Women (*n*=46,093)	Adjusted model a	Adjusted model b
City labor women’s empowerment, z score	0.97 (0.94, 0.99)	-
City Gini coefficient, z score	-	1.01 (0.98, 1.04)
Educational level (ref. University)	1.00	1.00
Secondary	1.16 (1.12, 1.20)	1.16 (1.12, 1.20)
Primary	1.30 (1.25, 1.35)	1.30 (1.25, 1.35)
Less than primary	1.23 (1.19, 1.27)	1.23 (1.20, 1.27)
Sub-city living conditions, z score	0.98 (0.97, 1.00)	0.98 (0.97, 1.00)
City GDP per capita (USD), z score	1.01 (1.00, 1.02)	1.01 (1.00, 1.02)
City population size, z score	0.98 (0.97, 0.99)	0.98 (0.97, 0.99)
Men (*n*= 33,329)	Adjusted model a	Adjusted model b
City labor women’s empowerment, z score	0.98 (0.95, 1.01)	-
Gini coefficient, z score	-	0.94 (0.90, 0.97)
Educational level (ref. University)	1.00	1.00
Secondary	1.00 (0.98, 1.03)	1.00 (0.98, 1.03)
Primary	0.94 (0.91, 0.97)	0.94 (0.91, 0.97)
Less than primary	0.88 (0.85, 0.91)	0.88 (0.85, 0.91)
Sub-city living conditions, z score	0.99 (0.97, 1.00)	0.99 (0.98, 1.01)
City GDP per capita (USD), z score	1.02 (1.01, 1.02)	1.02 (1.01, 1.02)
City population size, z score	0.99 (0.98, 1.00)	1.00 (0.99, 1.01)

Adjusted model a includes labor women’s empowerment; adjusted model b includes Gini coefficient; *PRRs*, prevalence rate ratios for each SD higher value of the predictor; *GDP*, gross domestic product. Multilevel structure: individuals nested within sub-cities, nested within cities. Analyses are adjusted by age, individual education, sub-city living conditions, city GDP per capita, city population size, and country

## Data Availability

When the data can be made public without violating confidentiality—once the SALURBAL project ends—it will be placed in a public repository as required by the funder (Wellcome Trust).
