# Supplementary material for: Are Women’s Empowerment and Income Inequality Associated with Excess Weight in Latin American Cities?
Source: J Urban Health. 2022 Nov 10;99(6):1091–103. doi: 10.1007/s11524-022-00689-5 (PMC7613896; doi:10.1007/s11524-022-00689-5)
Supplement: Supplementary file 1 — Supplementary file1 (DOCX 14 KB) [file 11524_2022_689_MOESM1_ESM.docx]

**Supplementary material**

**Table SI** Health survey and census data years for each country.

| Country | Health Survey data | Census data |
| --- | --- | --- |
| Argentina | 2013 | 2010 |
| Brazil | 2013 | 2010 |
| Chile | 2010 | 2002 |
| Colombia | 2007 | 2005 |
| El Salvador | 2014 | 2007 |
| Guatemala | 2002 | 2002 |
| Mexico | 2012 | 2010 |
| Peru | 2016 | 2017 |

**Table SII** Study variables by the level of analysis.

| ***Variable*** | ***Type*** | ***Level of analysis*** |
| --- | --- | --- |
| Overweight/Obesity | Outcome | Individual |
| Education | Effect-modifier | Individual |
| Age | Covariate | Individual |
| Gender | Stratification | Individual |
| Living conditions (composite index) | Effect-modifier | Sub-city |
| Labor women’s empowerment | Exposure | City |
| Gini coefficient | Exposure | City |
| Population size | Covariate | City |
| GDP per capita | Covariate | City |
| Country | Covariate | City |
